# Supplementary material for: Association of Health Disparities with Glioblastoma Treatment and Outcomes: Insights from a 15-Year National Cohort (2005–2020)
Source: Brain Sci. 2025 May 23;15(6):556. doi: 10.3390/brainsci15060556 (PMC12191283; doi:10.3390/brainsci15060556)

Supplementary Tables:

Table S1: ICD-O-3 Coding………………………………………………………………………………………….2

Table S2. Full Multivariable Model Assessing Sociodemographic and Clinical Predictors of Combined Sx+RT+Chemo……………….3

Table S3. Multivariable Logistic Regression: Reduced Model Assessing Sociodemographic Predictors of Receiving Combined Sx+RT+Chemo………………………………………………………………………………………………5

Table S4. Cox Model Stratified by Histology, Facility, Age, Insurance, Charlson–Deyo Score, and Location………………………....6

Table S5. Cox Model Stratified by Histology…………………………………………………………………………….7

Table S6. Cox Model Stratified by Facility Type……………………………………………………………………...9

Table S7. Cox Model Stratified by Age Group………………………………………………………………………….11

Table S8. Cox Model Stratified by Insurance Status…………………………………………………………………13

Table S9. Cox Model Stratified by Charlson–Deyo Comorbidity Score………………………………………………......15

Table S10. Cox Model Stratified by Geographic Location…………………………………………………………...17

Table S11. Multivariable Log-Normal Accelerated Failure Time Model for Overall Survival………………………………...19

Table S12. Multivariable Log-Logistic Accelerated Failure Time Model for Overall Survival……………………………………..22

Supplementary Figures:

Supplementary Figure S1. Scaled Schoenfeld Residual Plots for Race, Insurance, and Laterality……………………………………24

Supplementary Figure S2: Scaled Schoenfeld Residual Plots for Urbanicity (Metro/Non-Metro) and Location (Region)…………..25

Supplementary Figure S3. Scaled Schoenfeld Residual Plots for Age, Treatment, and Diagnosis Year…………………………...26

Table S1: ICD-O-3 Coding.

Group	ICD-O-3 Code	Type	Description
1	9440/3	Histology	Glioblastoma, NOS
2	9441/3	Histology	Giant cell glioblastoma
3	9442/3	Histology	Gliosarcoma
4	9445/3	Histology	Glioblastoma, IDH-mutant
5	C71.0	Primary Site	Cerebrum
6	C71.1	Primary Site	Frontal lobe
7	C71.2	Primary Site	Temporal lobe
8	C71.3	Primary Site	Parietal lobe
9	C71.4	Primary Site	Occipital lobe
10	C71.5	Primary Site	Ventricle
11	C71.6	Primary Site	Cerebellum
12	C71.7	Primary Site	Brainstem
13	C71.8	Primary Site	Overlapping lesion of brain
14	C71.9	Primary Site	Brain, NOS

Table S2: Full Multivariable Model Assessing Sociodemographic and Clinical Predictors of Combined Sx+RT+Chemo.

In the full multivariable model assessing predictors of receiving combined Sx+RT+Chemo, several sociodemographic differences were observed. Patients aged ≥65, women, and individuals identifying as non-White had significantly lower odds of receiving this treatment combination. Lower income, residence in areas with lower educational attainment (defined as ≥9.1% of adults without a high school diploma), treatment at community or network facilities, and government or no insurance were also independently associated with reduced likelihood of receiving combined treatment. Patients residing in the South, Midwest, or West were less likely to receive combination therapy compared to those in the Northeast. Findings should be interpreted with caution due to the observational design and potential for residual confounding.

**Dependent: Combined Sx+RT+Chemo**	**Levels**	**No**	**Yes**	**OR (univariable)**	**OR (multivariable)**
Age	<65 years	19170 (36.2)	33726 (63.8)	-	-
	≥65 years	33879 (57.4)	25180 (42.6)	0.42 (0.41-0.43, p<0.001)	0.52 (0.50-0.53, p<0.001)
Sex	Male	29566 (45.9)	34826 (54.1)	-	-
	Female	23483 (49.4)	24080 (50.6)	0.87 (0.85-0.89, p<0.001)	0.90 (0.88-0.92, p<0.001)
Race	White	48218 (47.2)	53952 (52.8)	-	-
	Non-white	4831 (49.4)	4954 (50.6)	0.92 (0.88-0.96, p<0.001)	0.90 (0.86-0.94, p<0.001)
Facility	Academic	19909 (43.0)	26377 (57.0)	-	-
	Community	22631 (51.9)	20999 (48.1)	0.70 (0.68-0.72, p<0.001)	0.78 (0.76-0.80, p<0.001)
	Network	10509 (47.7)	11530 (52.3)	0.83 (0.80-0.86, p<0.001)	0.87 (0.84-0.90, p<0.001)
Income group	<$74,063	33581 (50.1)	33410 (49.9)	-	-
	≥$74,063	19468 (43.3)	25496 (56.7)	1.32 (1.29-1.35, p<0.001)	1.15 (1.12-1.19, p<0.001)
No high school rate	≥9.1%	25392 (50.6)	24744 (49.4)	-	-
	<9.1%	27657 (44.7)	34162 (55.3)	1.27 (1.24-1.30, p<0.001)	1.14 (1.11-1.17, p<0.001)
Charlson–Deyo Score	0	34699 (44.6)	43167 (55.4)	-	-
	1	10073 (51.3)	9556 (48.7)	0.76 (0.74-0.79, p<0.001)	0.86 (0.83-0.89, p<0.001)
	2	4914 (55.1)	4012 (44.9)	0.66 (0.63-0.69, p<0.001)	0.74 (0.71-0.78, p<0.001)
	3	3363 (60.8)	2171 (39.2)	0.52 (0.49-0.55, p<0.001)	0.62 (0.59-0.66, p<0.001)
Insurance	Private	16516 (36.0)	29396 (64.0)	-	-
	Government	34727 (55.5)	27887 (44.5)	0.45 (0.44-0.46, p<0.001)	0.74 (0.71-0.76, p<0.001)

	Uninsured	1806 (52.7)	1623 (47.3)	0.50 (0.47-0.54, p<0.001)	0.54 (0.50-0.58, p<0.001)
Location	Northeast	18209 (44.2)	22976 (55.8)	-	-
	South	15740 (49.3)	16164 (50.7)	0.81 (0.79-0.84, p<0.001)	0.88 (0.85-0.91, p<0.001)
	Midwest	11345 (49.9)	11372 (50.1)	0.79 (0.77-0.82, p<0.001)	0.83 (0.80-0.86, p<0.001)
	West	7755 (48.0)	8394 (52.0)	0.86 (0.83-0.89, p<0.001)	0.85 (0.81-0.88, p<0.001)

Abbreviations: CI = Confidence Interval; OR = Odds Ratio; RT = Radiation Therapy; Sx = Surgery; Chemo = Chemotherapy

Table S3: Multivariable Logistic Regression: Reduced Model Assessing Sociodemographic Predictors of Receiving Combined Sx+RT+Chemo.

In the reduced model limited to key sociodemographic and insurance-related variables, several differences in treatment receipt remained significant. Female sex, lower income, residence in less-educated areas (defined as ≥9.1% of adults without a high school diploma), and government or no insurance were associated with lower odds of receiving multi-modality therapy. Race and ethnicity were no longer significant after adjustment for these factors. These findings reflect sociodemographic variation in treatment patterns. Interpretations should be made cautiously given the observational nature of the data and potential residual confounding.

**Dependent: Combined Sx+RT+Chemo**	**Levels**	**No**	**Yes**	**OR (univariable)**	**OR (multivariable)**
Sex	Male	29566 (45.9)	34826 (54.1)	-	-
	Female	23483 (49.4)	24080 (50.6)	0.87 (0.85-0.89, p<0.001)	0.89 (0.87-0.91, p<0.001)
Race	White	48218 (47.2)	53952 (52.8)	-	-
	Non-white	4831 (49.4)	4954 (50.6)	0.92 (0.88-0.96, p<0.001)	0.96 (0.92-1.00, p=0.082)
Ethnicity	Non-Hispanic	50088 (47.3)	55897 (52.7)	-	-
	Hispanic	2961 (49.6)	3009 (50.4)	0.91 (0.86-0.96, p<0.001)	0.97 (0.92-1.03, p=0.352)
Income group	<$74,063	33581 (50.1)	33410 (49.9)	-	-
	≥$74,063	19468 (43.3)	25496 (56.7)	1.32 (1.29-1.35, p<0.001)	1.16 (1.13-1.20, p<0.001)
No high school rate	≥9.1%	25392 (50.6)	24744 (49.4)	-	-
	<9.1%	27657 (44.7)	34162 (55.3)	1.27 (1.24-1.30, p<0.001)	1.13 (1.10-1.16, p<0.001)
Insurance	Private	16516 (36.0)	29396 (64.0)	-	-
	Government	34727 (55.5)	27887 (44.5)	0.45 (0.44-0.46, p<0.001)	0.46 (0.45-0.47, p<0.001)
	Uninsured	1806 (52.7)	1623 (47.3)	0.50 (0.47-0.54, p<0.001)	0.53 (0.50-0.57, p<0.001)

Abbreviations: CI = Confidence Interval; Chemo = Chemotherapy; OR = Odds Ratio; RT = Radiation Therapy; Sx = Surgery

Table S4. Cox Model with 6 Variables Stratified by Histology, Facility, Age, Insurance, Charlson–Deyo Score, and Location.

Stratification by key clinical variables (histology, facility type, age, insurance, comorbidity burden, and geographic location) was performed to account for potential non-proportional hazards across patient subgroups. Adjusted hazard estimates remained stable across these stratifications. Interpretations should be made cautiously given the observational nature of the data and the possibility of residual confounding.

Variable	Levels	n (%)	HR (univariable)	HR (multivariable)
Treatment	Sx+RT+Chemo	58906 (52.6)	-	-
	RT+Chemo	12102 (10.8)	1.74 (1.70-1.77, p<0.001)	1.66 (1.63-1.70, p<0.001)
	RT+Sx	5890 (5.3)	1.69 (1.64-1.74, p<0.001)	1.58 (1.53-1.62, p<0.001)
	Sx	16777 (15.0)	2.47 (2.43-2.52, p<0.001)	2.38 (2.33-2.42, p<0.001)
	Other	6411 (5.7)	2.12 (2.06-2.18, p<0.001)	2.02 (1.96-2.07, p<0.001)
	No treatment	11869 (10.6)	5.07 (4.96-5.18, p<0.001)	4.40 (4.31-4.50, p<0.001)
Sex	Male	64392 (57.5)	-	-
	Female	47563 (42.5)	0.99 (0.98-1.00, p=0.058)	0.94 (0.93-0.96, p<0.001)
Race	White	102170 (91.3)	-	-
	Non-white	9785 (8.7)	0.83 (0.81-0.85, p<0.001)	0.79 (0.77-0.81, p<0.001)
Ethnicity	Non-Hispanic	105985 (94.7)	-	-
	Hispanic	5970 (5.3)	0.80 (0.77-0.82, p<0.001)	0.77 (0.74-0.79, p<0.001)
Income group	<$74,063	66991 (59.8)	-	-
	≥$74,063	44964 (40.2)	0.83 (0.82-0.84, p<0.001)	0.87 (0.85-0.88, p<0.001)
No high school rate	≥9.1%	50136 (44.8)	-	-
	<9.1%	61819 (55.2)	0.94 (0.92-0.95, p<0.001)	1.04 (1.03-1.06, p<0.001)
Region	Metro	94101 (84.1)	-	-
	Non-metro	17854 (15.9)	1.16 (1.14-1.18, p<0.001)	1.05 (1.03-1.07, p<0.001)
Laterality	Unilateral	82791 (74.0)	-	-
	Bilateral/midline	29164 (26.0)	1.24 (1.23-1.26, p<0.001)	1.13 (1.11-1.14, p<0.001)

Abbreviations: Chemo = Chemotherapy; HR = Hazards Ratio; RT = Radiation Therapy; Sx = Surgery

Table S5. Cox Model Stratified by Histology.

Stratification by tumor histology (e.g., glioblastoma NOS, giant cell GBM, gliosarcoma, IDH-mutant GBM) was performed to account for heterogeneity in survival outcomes. Patients with IDH-mutant glioblastoma demonstrated more favorable survival compared to conventional GBM, in line with established prognostic trends. Model adjustments for histologic subtype did not significantly alter hazard estimates across other variables.

Variable	Levels	n (%)	HR (univariable)	HR (multivariable)
**Treatment**	Sx+RT+Chemo	58906 (52.6)	-	-
	RT+Chemo	12102 (10.8)	1.74 (1.70-1.77, p<0.001)	1.66 (1.62-1.69, p<0.001)
	RT+Sx	5890 (5.3)	1.69 (1.64-1.74, p<0.001)	1.60 (1.55-1.64, p<0.001)
	Sx	16777 (15.0)	2.47 (2.43-2.52, p<0.001)	2.36 (2.32-2.40, p<0.001)
	Other	6411 (5.7)	2.12 (2.06-2.18, p<0.001)	2.01 (1.96-2.07, p<0.001)
	No treatment	11869 (10.6)	5.07 (4.96-5.18, p<0.001)	4.38 (4.28-4.47, p<0.001)
**Age**	<65 years	52896 (47.2)	-	-
	≥65 years	59059 (52.8)	1.82 (1.80-1.85, p<0.001)	1.43 (1.41-1.46, p<0.001)
**Sex**	Male	64392 (57.5)	-	-
	Female	47563 (42.5)	0.99 (0.98-1.00, p=0.058)	0.95 (0.94-0.96, p<0.001)
**Race**	White	102170 (91.3)	-	-
	Non-white	9785 (8.7)	0.83 (0.81-0.85, p<0.001)	0.78 (0.76-0.80, p<0.001)
**Ethnicity**	Non-Hispanic	105985 (94.7)	-	-
	Hispanic	5970 (5.3)	0.80 (0.77-0.82, p<0.001)	0.75 (0.73-0.77, p<0.001)
**Facility**	Academic	46286 (41.3)	-	-
	Community	43630 (39.0)	1.24 (1.23-1.26, p<0.001)	1.09 (1.07-1.10, p<0.001)
	Network	22039 (19.7)	1.18 (1.16-1.20, p<0.001)	1.12 (1.11-1.14, p<0.001)
**Income group**	<$74,063	66991 (59.8)	-	-
	≥$74,063	44964 (40.2)	0.83 (0.82-0.84, p<0.001)	0.86 (0.85-0.87, p<0.001)
**No high school rate**	≥9.1%	50136 (44.8)	-	-
	<9.1%	61819 (55.2)	0.94 (0.92-0.95, p<0.001)	1.05 (1.04-1.07, p<0.001)
**Region**	Metro	94101 (84.1)	-	-

	Non-metro	17854 (15.9)	1.16 (1.14-1.18, p<0.001)	1.06 (1.04-1.08, p<0.001)
**Charlson–Deyo Score**	0	77866 (69.6)	-	-
	1	19629 (17.5)	1.28 (1.26-1.31, p<0.001)	1.18 (1.16-1.20, p<0.001)
	2	8926 (8.0)	1.41 (1.38-1.45, p<0.001)	1.28 (1.25-1.31, p<0.001)
	3	5534 (4.9)	1.67 (1.62-1.72, p<0.001)	1.44 (1.40-1.48, p<0.001)
**Insurance**	Private	45912 (41.0)	-	-
	Government	62614 (55.9)	1.67 (1.65-1.69, p<0.001)	1.15 (1.13-1.17, p<0.001)
	Uninsured	3429 (3.1)	1.14 (1.09-1.18, p<0.001)	1.01 (0.97-1.05, p=0.623)
**Location**	Northeast	41185 (36.8)	-	-
	South	31904 (28.5)	1.11 (1.09-1.13, p<0.001)	1.04 (1.03-1.06, p<0.001)
	Midwest	22717 (20.3)	1.04 (1.02-1.05, p<0.001)	0.95 (0.93-0.96, p<0.001)
	West	16149 (14.4)	1.04 (1.02-1.06, p<0.001)	1.06 (1.04-1.09, p<0.001)
**Laterality**	Unilateral	82791 (74.0)	-	-
	Bilateral/midline	29164 (26.0)	1.24 (1.23-1.26, p<0.001)	1.12 (1.11-1.14, p<0.001)

Abbreviations: Chemo = Chemotherapy; HR = Hazards Ratio; RT = Radiation Therapy; Sx = Surgery

Table S6. Cox Model Stratified by Facility.

Stratification by facility type (academic, community, network) was performed to assess whether survival differences varied by treatment setting. Hazard ratios were generally consistent across facility types, with minor attenuation observed at community and network centers.

Variable	Levels	n (%)	HR (univariable)	HR (multivariable)
**Treatment**	Sx+RT+Chemo	58906 (52.6)	-	-
	RT+Chemo	12102 (10.8)	1.74 (1.70-1.77, p<0.001)	1.65 (1.62-1.69, p<0.001)
	RT+Sx	5890 (5.3)	1.69 (1.64-1.74, p<0.001)	1.60 (1.56-1.65, p<0.001)
	Sx	16777 (15.0)	2.47 (2.43-2.52, p<0.001)	2.36 (2.32-2.40, p<0.001)
	Other	6411 (5.7)	2.12 (2.06-2.18, p<0.001)	2.02 (1.96-2.07, p<0.001)
	No treatment	11869 (10.6)	5.07 (4.96-5.18, p<0.001)	4.38 (4.29-4.47, p<0.001)
**Age**	<65 years	52896 (47.2)	-	-
	≥65 years	59059 (52.8)	1.82 (1.80-1.85, p<0.001)	1.43 (1.41-1.46, p<0.001)
**Sex**	Male	64392 (57.5)	-	-
	Female	47563 (42.5)	0.99 (0.98-1.00, p=0.058)	0.95 (0.94-0.96, p<0.001)
**Race**	White	102170 (91.3)	-	-
	Non-white	9785 (8.7)	0.83 (0.81-0.85, p<0.001)	0.78 (0.76-0.80, p<0.001)
**Ethnicity**	Non-Hispanic	105985 (94.7)	-	-
	Hispanic	5970 (5.3)	0.80 (0.77-0.82, p<0.001)	0.75 (0.73-0.77, p<0.001)
**Income group**	<$74,063	66991 (59.8)	-	-
	≥$74,063	44964 (40.2)	0.83 (0.82-0.84, p<0.001)	0.86 (0.85-0.87, p<0.001)
**No high school rate**	≥9.1%	50136 (44.8)	-	-
	<9.1%	61819 (55.2)	0.94 (0.92-0.95, p<0.001)	1.05 (1.04-1.07, p<0.001)
**Region**	Metro	94101 (84.1)	-	-
	Non-metro	17854 (15.9)	1.16 (1.14-1.18, p<0.001)	1.06 (1.04-1.08, p<0.001)
**Charlson–Deyo Score**	0	77866 (69.6)	-	-
	1	19629 (17.5)	1.28 (1.26-1.31, p<0.001)	1.18 (1.16-1.20, p<0.001)
	2	8926 (8.0)	1.41 (1.38-1.45, p<0.001)	1.28 (1.25-1.31, p<0.001)
	3	5534 (4.9)	1.67 (1.62-1.72, p<0.001)	1.44 (1.40-1.48, p<0.001)
**Insurance**	Private	45912 (41.0)	-	-

	Government	62614 (55.9)	1.67 (1.65-1.69, p<0.001)	1.15 (1.13-1.17, p<0.001)
	Uninsured	3429 (3.1)	1.14 (1.09-1.18, p<0.001)	1.01 (0.97-1.05, p=0.617)
**Location**	Northeast	41185 (36.8)	-	-
	South	31904 (28.5)	1.11 (1.09-1.13, p<0.001)	1.04 (1.03-1.06, p<0.001)
	Midwest	22717 (20.3)	1.04 (1.02-1.05, p<0.001)	0.95 (0.93-0.96, p<0.001)
	West	16149 (14.4)	1.04 (1.02-1.06, p<0.001)	1.07 (1.04-1.09, p<0.001)
**Histology**	Glioblastoma, NOS	108426 (96.8)	-	-
	Giant cell glioblastoma	766 (0.7)	0.72 (0.67-0.78, p<0.001)	0.81 (0.75-0.88, p<0.001)
	Gliosarcoma	2377 (2.1)	0.92 (0.88-0.96, p<0.001)	1.03 (0.99-1.07, p=0.175)
	Glioblastoma, IDH-mutant	386 (0.3)	0.45 (0.40-0.52, p<0.001)	0.56 (0.49-0.64, p<0.001)
**Laterality**	Unilateral	82791 (74.0)	-	-
	Bilateral/midline	29164 (26.0)	1.24 (1.23-1.26, p<0.001)	1.12 (1.11-1.14, p<0.001)

Abbreviations: Sx: Surgery; RT: Radiation Therapy; Chemo: Chemotherapy; HR: Hazard Ratio; CI: Confidence Interval; NOS: Not Otherwise Specified; IDH: Isocitrate Dehydrogenase; GBM: Glioblastoma Multiforme; Metro: Metropolitan area; Non-metro: Non-metropolitan area; HS: High School; Charlson–Deyo Score: Comorbidity index based on ICD codes; n (%): Frequency and percentage.

Table S7. Cox Model Stratified by Age.

Stratification by age group (<65 vs. ≥65 years) was performed to examine potential variation in survival associations across different age populations. Although older patients had higher overall mortality, hazard ratio patterns remained generally consistent across age strata.

Variable	Levels	n (%)	HR (univariable)	HR (multivariable)
**Treatment**	Sx+RT+Chemo	58906 (52.6)	-	-
	RT+Chemo	12102 (10.8)	1.74 (1.70-1.77, p<0.001)	1.65 (1.62-1.69, p<0.001)
	RT+Sx	5890 (5.3)	1.69 (1.64-1.74, p<0.001)	1.57 (1.52-1.61, p<0.001)
	Sx	16777 (15.0)	2.47 (2.43-2.52, p<0.001)	2.34 (2.30-2.39, p<0.001)
	Other	6411 (5.7)	2.12 (2.06-2.18, p<0.001)	1.99 (1.94-2.05, p<0.001)
	No treatment	11869 (10.6)	5.07 (4.96-5.18, p<0.001)	4.32 (4.22-4.41, p<0.001)
**Sex**	Male	64392 (57.5)	-	-
	Female	47563 (42.5)	0.99 (0.98-1.00, p=0.058)	0.94 (0.93-0.96, p<0.001)
**Race**	White	102170 (91.3)	-	-
	Non-white	9785 (8.7)	0.83 (0.81-0.85, p<0.001)	0.79 (0.77-0.80, p<0.001)
**Ethnicity**	Non-Hispanic	105985 (94.7)	-	-
	Hispanic	5970 (5.3)	0.80 (0.77-0.82, p<0.001)	0.75 (0.73-0.77, p<0.001)
**Facility**	Academic	46286 (41.3)	-	-
	Community	43630 (39.0)	1.24 (1.23-1.26, p<0.001)	1.09 (1.07-1.10, p<0.001)
	Network	22039 (19.7)	1.18 (1.16-1.20, p<0.001)	1.12 (1.10-1.14, p<0.001)
**Income group**	<$74,063	66991 (59.8)	-	-
	≥$74,063	44964 (40.2)	0.83 (0.82-0.84, p<0.001)	0.86 (0.85-0.87, p<0.001)
**No high school rate**	≥9.1%	50136 (44.8)	-	-
	<9.1%	61819 (55.2)	0.94 (0.92-0.95, p<0.001)	1.05 (1.03-1.06, p<0.001)
**Region**	Metro	94101 (84.1)	-	-
	Non-metro	17854 (15.9)	1.16 (1.14-1.18, p<0.001)	1.05 (1.04-1.07, p<0.001)
**Charlson–Deyo Score**	0	77866 (69.6)	-	-
	1	19629 (17.5)	1.28 (1.26-1.31, p<0.001)	1.18 (1.16-1.20, p<0.001)
	2	8926 (8.0)	1.41 (1.38-1.45, p<0.001)	1.28 (1.25-1.31, p<0.001)

	3	5534 (4.9)	1.67 (1.62-1.72, p<0.001)	1.43 (1.39-1.47, p<0.001)
**Insurance**	Private	45912 (41.0)	-	-
	Government	62614 (55.9)	1.67 (1.65-1.69, p<0.001)	1.16 (1.14-1.18, p<0.001)
	Uninsured	3429 (3.1)	1.14 (1.09-1.18, p<0.001)	1.03 (0.99-1.07, p=0.192)
**Location**	Northeast	41185 (36.8)	-	-
	South	31904 (28.5)	1.11 (1.09-1.13, p<0.001)	1.04 (1.02-1.06, p<0.001)
	Midwest	22717 (20.3)	1.04 (1.02-1.05, p<0.001)	0.95 (0.93-0.96, p<0.001)
	West	16149 (14.4)	1.04 (1.02-1.06, p<0.001)	1.06 (1.04-1.08, p<0.001)
**Histology**	Glioblastoma, NOS	108426 (96.8)	-	-
	Giant cell glioblastoma	766 (0.7)	0.72 (0.67-0.78, p<0.001)	0.81 (0.75-0.87, p<0.001)
	Gliosarcoma	2377 (2.1)	0.92 (0.88-0.96, p<0.001)	1.03 (0.99-1.08, p=0.169)
	Glioblastoma, IDH-mutant	386 (0.3)	0.45 (0.40-0.52, p<0.001)	0.56 (0.49-0.63, p<0.001)
**Laterality**	Unilateral	82791 (74.0)	-	-
	Bilateral/midline	29164 (26.0)	1.24 (1.23-1.26, p<0.001)	1.13 (1.11-1.14, p<0.001)

Abbreviations: Sx: Surgery; RT: Radiation Therapy; Chemo: Chemotherapy; HR: Hazard Ratio; CI: Confidence Interval; NOS: Not Otherwise Specified; IDH: Isocitrate Dehydrogenase; GBM: Glioblastoma Multiforme; Metro: Metropolitan area; Non-metro: Non-metropolitan area; HS: High School; Charlson–Deyo Score: Comorbidity index based on ICD codes; n (%): Frequency and percentage.

Table S8. Cox Model Stratified by Insurance.

Stratification by insurance type (private, government, uninsured) was conducted to explore potential differences in survival associations across payer categories. Results remained broadly consistent across strata, though slight attenuation of hazard ratios was noted among uninsured patients, which may reflect differential access to supportive care or other unmeasured factors.

Variable	Levels	n (%)	HR (univariable)	HR (multivariable)
**Treatment**	Sx+RT+Chemo	58906 (52.6)	-	-
	RT+Chemo	12102 (10.8)	1.74 (1.70-1.77, p<0.001)	1.66 (1.62-1.69, p<0.001)
	RT+Sx	5890 (5.3)	1.69 (1.64-1.74, p<0.001)	1.58 (1.53-1.62, p<0.001)
	Sx	16777 (15.0)	2.47 (2.43-2.52, p<0.001)	2.35 (2.30-2.39, p<0.001)
	Other	6411 (5.7)	2.12 (2.06-2.18, p<0.001)	2.00 (1.95-2.06, p<0.001)
	No treatment	11869 (10.6)	5.07 (4.96-5.18, p<0.001)	4.35 (4.26-4.44, p<0.001)
**Age**	<65 years	52896 (47.2)	-	-
	≥65 years	59059 (52.8)	1.82 (1.80-1.85, p<0.001)	1.44 (1.42-1.47, p<0.001)
**Sex**	Male	64392 (57.5)	-	-
	Female	47563 (42.5)	0.99 (0.98-1.00, p=0.058)	0.95 (0.93-0.96, p<0.001)
**Race**	White	102170 (91.3)	-	-
	Non-white	9785 (8.7)	0.83 (0.81-0.85, p<0.001)	0.79 (0.77-0.80, p<0.001)
**Ethnicity**	Non-Hispanic	105985 (94.7)	-	-
	Hispanic	5970 (5.3)	0.80 (0.77-0.82, p<0.001)	0.76 (0.74-0.78, p<0.001)
**Facility**	Academic	46286 (41.3)	-	-
	Community	43630 (39.0)	1.24 (1.23-1.26, p<0.001)	1.09 (1.07-1.10, p<0.001)
	Network	22039 (19.7)	1.18 (1.16-1.20, p<0.001)	1.12 (1.10-1.14, p<0.001)
**Income group**	<$74,063	66991 (59.8)	-	-
	≥$74,063	44964 (40.2)	0.83 (0.82-0.84, p<0.001)	0.86 (0.85-0.87, p<0.001)
**No high school rate**	≥9.1%	50136 (44.8)	-	-
	<9.1%	61819 (55.2)	0.94 (0.92-0.95, p<0.001)	1.05 (1.03-1.06, p<0.001)
**Region**	Metro	94101 (84.1)	-	-
	Non-metro	17854 (15.9)	1.16 (1.14-1.18, p<0.001)	1.05 (1.04-1.07, p<0.001)
**Charlson–Deyo Score**	0	77866 (69.6)	-	-

	1	19629 (17.5)	1.28 (1.26-1.31, p<0.001)	1.18 (1.16-1.20, p<0.001)
	2	8926 (8.0)	1.41 (1.38-1.45, p<0.001)	1.28 (1.25-1.31, p<0.001)
	3	5534 (4.9)	1.67 (1.62-1.72, p<0.001)	1.43 (1.39-1.47, p<0.001)
**Location**	Northeast	41185 (36.8)	-	-
	South	31904 (28.5)	1.11 (1.09-1.13, p<0.001)	1.04 (1.03-1.06, p<0.001)
	Midwest	22717 (20.3)	1.04 (1.02-1.05, p<0.001)	0.95 (0.93-0.96, p<0.001)
	West	16149 (14.4)	1.04 (1.02-1.06, p<0.001)	1.06 (1.04-1.09, p<0.001)
**Histology**	Glioblastoma, NOS	108426 (96.8)	-	-
	Giant cell glioblastoma	766 (0.7)	0.72 (0.67-0.78, p<0.001)	0.80 (0.74-0.87, p<0.001)
	Gliosarcoma	2377 (2.1)	0.92 (0.88-0.96, p<0.001)	1.03 (0.99-1.08, p=0.122)
	Glioblastoma, IDH-mutant	386 (0.3)	0.45 (0.40-0.52, p<0.001)	0.56 (0.49-0.64, p<0.001)
**Laterality**	Unilateral	82791 (74.0)	-	-
	Bilateral/Midline	29164 (26.0)	1.24 (1.23-1.26, p<0.001)	1.13 (1.11-1.14, p<0.001)

Abbreviations: Sx: Surgery; RT: Radiation Therapy; Chemo: Chemotherapy; HR: Hazard Ratio; CI: Confidence Interval; NOS: Not Otherwise Specified; IDH: Isocitrate Dehydrogenase; GBM: Glioblastoma Multiforme; Metro: Metropolitan area; Non-metro: Non-metropolitan area; HS: High School; Charlson–Deyo Score: Comorbidity index based on ICD codes; n (%): Frequency and percentage.

Table S9. Cox Model Stratified by Charlson–Deyo Score.

Stratification by comorbidity burden (Charlson–Deyo Score 0–3) was conducted to assess whether survival associations varied by comorbid condition severity. Results remained generally consistent across strata, with minimal deviation in hazard ratio estimates across comorbidity groups. Interpretations should be made cautiously given potential residual confounding and the observational nature of the dataset.

Variable	Levels	n (%)	HR (univariable)	HR (multivariable)
**Treatment**	Sx+RT+Chemo	58906 (52.6)	-	-
	RT+Chemo	12102 (10.8)	1.74 (1.70-1.77, p<0.001)	1.66 (1.62-1.69, p<0.001)
	RT+Sx	5890 (5.3)	1.69 (1.64-1.74, p<0.001)	1.59 (1.55-1.64, p<0.001)
	Sx	16777 (15.0)	2.47 (2.43-2.52, p<0.001)	2.35 (2.31-2.39, p<0.001)
	Other	6411 (5.7)	2.12 (2.06-2.18, p<0.001)	2.01 (1.96-2.07, p<0.001)
	No treatment	11869 (10.6)	5.07 (4.96-5.18, p<0.001)	4.36 (4.27-4.46, p<0.001)
**Age**	<65 years	52896 (47.2)	-	-
	≥65 years	59059 (52.8)	1.82 (1.80-1.85, p<0.001)	1.43 (1.41-1.46, p<0.001)
**Sex**	Male	64392 (57.5)	-	-
	Female	47563 (42.5)	0.99 (0.98-1.00, p=0.058)	0.95 (0.94-0.96, p<0.001)
**Race**	White	102170 (91.3)	-	-
	Non-white	9785 (8.7)	0.83 (0.81-0.85, p<0.001)	0.78 (0.76-0.80, p<0.001)
**Ethnicity**	Non-Hispanic	105985 (94.7)	-	-
	Hispanic	5970 (5.3)	0.80 (0.77-0.82, p<0.001)	0.75 (0.73-0.77, p<0.001)
**Facility**	Academic	46286 (41.3)	-	-
	Community	43630 (39.0)	1.24 (1.23-1.26, p<0.001)	1.09 (1.07-1.10, p<0.001)
	Network	22039 (19.7)	1.18 (1.16-1.20, p<0.001)	1.12 (1.11-1.14, p<0.001)
**Income group**	<$74,063	66991 (59.8)	-	-
	≥$74,063	44964 (40.2)	0.83 (0.82-0.84, p<0.001)	0.86 (0.85-0.87, p<0.001)
**No high school rate**	≥9.1%	50136 (44.8)	-	-
	<9.1%	61819 (55.2)	0.94 (0.92-0.95, p<0.001)	1.05 (1.04-1.07, p<0.001)
**Region**	Metro	94101 (84.1)	-	-
	Non-metro	17854 (15.9)	1.16 (1.14-1.18, p<0.001)	1.06 (1.04-1.08, p<0.001)

**Insurance**	Private	45912 (41.0)	-	-
	Government	62614 (55.9)	1.67 (1.65-1.69, p<0.001)	1.15 (1.13-1.17, p<0.001)
	Uninsured	3429 (3.1)	1.14 (1.09-1.18, p<0.001)	1.01 (0.97-1.05, p=0.564)
**Location**	Northeast	41185 (36.8)	-	-
	South	31904 (28.5)	1.11 (1.09-1.13, p<0.001)	1.04 (1.03-1.06, p<0.001)
	Midwest	22717 (20.3)	1.04 (1.02-1.05, p<0.001)	0.95 (0.93-0.96, p<0.001)
	West	16149 (14.4)	1.04 (1.02-1.06, p<0.001)	1.06 (1.04-1.08, p<0.001)
**Histology**	Glioblastoma, NOS	108426 (96.8)	-	-
	Giant cell glioblastoma	766 (0.7)	0.72 (0.67-0.78, p<0.001)	0.81 (0.75-0.87, p<0.001)
	Gliosarcoma	2377 (2.1)	0.92 (0.88-0.96, p<0.001)	1.03 (0.99-1.07, p=0.181)
	Glioblastoma, IDH-mutant	386 (0.3)	0.45 (0.40-0.52, p<0.001)	0.56 (0.49-0.64, p<0.001)
**Laterality**	Unilateral	82791 (74.0)	-	-
	Bilateral/midline	29164 (26.0)	1.24 (1.23-1.26, p<0.001)	1.12 (1.11-1.14, p<0.001)

Abbreviations: Sx: Surgery; RT: Radiation Therapy; Chemo: Chemotherapy; HR: Hazard Ratio; CI: Confidence Interval; NOS: Not Otherwise Specified; IDH: Isocitrate Dehydrogenase; GBM: Glioblastoma Multiforme; Metro: Metropolitan area; Non-metro: Non-metropolitan area; HS: High School; Charlson–Deyo Score: Comorbidity index based on ICD codes; n (%): Frequency and percentage.

Table S10. Cox Model Stratified by Location.

Stratification by geographic region (Northeast, South, Midwest, West) was performed to evaluate whether survival associations varied across U.S. regions. Minor regional differences in hazard ratios were observed; however, the overall associations remained stable, suggesting broad geographic consistency. Findings should be interpreted with caution given the observational nature of the dataset and potential residual confounding.

**Variable**	**Levels**	**n (%)**	**HR (univariable)**	**HR (multivariable)**
**Treatment**	Sx+RT+Chemo	58906 (52.6)	-	-
	RT+Chemo	12102 (10.8)	1.74 (1.70-1.77, p<0.001)	1.66 (1.62-1.69, p<0.001)
	RT+Sx	5890 (5.3)	1.69 (1.64-1.74, p<0.001)	1.60 (1.56-1.64, p<0.001)
	Sx	16777 (15.0)	2.47 (2.43-2.52, p<0.001)	2.36 (2.32-2.41, p<0.001)
	Other	6411 (5.7)	2.12 (2.06-2.18, p<0.001)	2.02 (1.96-2.07, p<0.001)
	No treatment	11869 (10.6)	5.07 (4.96-5.18, p<0.001)	4.39 (4.29-4.48, p<0.001)
**Age**	<65 years	52896 (47.2)	-	-
	≥65 years	59059 (52.8)	1.82 (1.80-1.85, p<0.001)	1.43 (1.41-1.46, p<0.001)
**Sex**	Male	64392 (57.5)	-	-
	Female	47563 (42.5)	0.99 (0.98-1.00, p=0.058)	0.95 (0.93-0.96, p<0.001)
**Race**	White	102170 (91.3)	-	-
	Non-white	9785 (8.7)	0.83 (0.81-0.85, p<0.001)	0.78 (0.76-0.80, p<0.001)
**Ethnicity**	Non-Hispanic	105985 (94.7)	-	-
	Hispanic	5970 (5.3)	0.80 (0.77-0.82, p<0.001)	0.75 (0.73-0.77, p<0.001)
**Facility**	Academic	46286 (41.3)	-	-
	Community	43630 (39.0)	1.24 (1.23-1.26, p<0.001)	1.09 (1.07-1.10, p<0.001)
	Network	22039 (19.7)	1.18 (1.16-1.20, p<0.001)	1.13 (1.11-1.14, p<0.001)
**Income group**	<$74,063	66991 (59.8)	-	-
	≥$74,063	44964 (40.2)	0.83 (0.82-0.84, p<0.001)	0.86 (0.85-0.87, p<0.001)
**No high school rate**	≥9.1%	50136 (44.8)	-	-
	<9.1%	61819 (55.2)	0.94 (0.92-0.95, p<0.001)	1.05 (1.04-1.07, p<0.001)
**Region**	Metro	94101 (84.1)	-	-
	Non-metro	17854 (15.9)	1.16 (1.14-1.18, p<0.001)	1.06 (1.04-1.08, p<0.001)

**Charlson–Deyo Score**	0	77866 (69.6)	-	-
	1	19629 (17.5)	1.28 (1.26-1.31, p<0.001)	1.18 (1.16-1.20, p<0.001)
	2	8926 (8.0)	1.41 (1.38-1.45, p<0.001)	1.28 (1.25-1.31, p<0.001)
	3	5534 (4.9)	1.67 (1.62-1.72, p<0.001)	1.44 (1.40-1.48, p<0.001)
**Insurance**	Private	45912 (41.0)	-	-
	Government	62614 (55.9)	1.67 (1.65-1.69, p<0.001)	1.15 (1.13-1.17, p<0.001)
	Uninsured	3429 (3.1)	1.14 (1.09-1.18, p<0.001)	1.01 (0.97-1.05, p=0.570)
**Histology**	Glioblastoma, NOS	108426 (96.8)	-	-
	Giant cell glioblastoma	766 (0.7)	0.72 (0.67-0.78, p<0.001)	0.81 (0.75-0.88, p<0.001)
	Gliosarcoma	2377 (2.1)	0.92 (0.88-0.96, p<0.001)	1.03 (0.99-1.07, p=0.173)
	Glioblastoma, IDH-mutant	386 (0.3)	0.45 (0.40-0.52, p<0.001)	0.56 (0.50-0.64, p<0.001)
**Laterality**	Unilateral	82791 (74.0)	-	-
	Bilateral/midline	29164 (26.0)	1.24 (1.23-1.26, p<0.001)	1.12 (1.11-1.14, p<0.001)

Abbreviations: Sx: Surgery; RT: Radiation Therapy; Chemo: Chemotherapy; HR: Hazard Ratio; CI: Confidence Interval; NOS: Not Otherwise Specified; IDH: Isocitrate Dehydrogenase; GBM: Glioblastoma Multiforme; Metro: Metropolitan area; Non-metro: Non-metropolitan area; HS: High School; Charlson–Deyo Score: Comorbidity index based on ICD codes; n (%): Frequency and percentage.

Table S11. Multivariable Log-Normal Accelerated Failure Time Model for Overall Survival.

Multivariable AFT model results are shown below with consistent listing of reference categories for each variable. Time ratios (TRs) >1 suggest prolonged survival time relative to the reference group, while TRs <1 indicate shorter survival duration.

Variable	Category	Time Ratio (TR)	95% CI	P-value
**Treatment**	Sx+RT+Chemo (Reference)	1.00	—	—
	RT+Chemo	0.55	0.54–0.56	<0.001
	RT+Surgery	0.56	0.55–0.57	<0.001
	Sx	0.26	0.25–0.26	<0.001
	Other	0.39	0.38–0.40	<0.001
	No treatment	0.14	0.14–0.15	<0.001
**Age**	<65 years (Reference)	1.00	—	—
	≥65 years	0.70	0.69–0.71	<0.001
**Sex**	Male (Reference)	1.00	—	—
	Female	1.06	1.05–1.07	<0.001
**Race**	White (Reference)	1.00	—	—
	Non-White	1.22	1.20–1.24	<0.001
**Ethnicity**	Non-Hispanic (Reference)	1.00	—	—
	Hispanic	1.26	1.22–1.29	<0.001
**Facility Type**	Academic (Reference)	1.00	—	—
	Community	0.89	0.88–0.90	<0.001
	Network	0.89	0.88–0.90	<0.001

**Income Group**	<$74,063 (Reference)	1.00	—	—
	≥$74,063	1.14	1.13–1.15	<0.001
**Education**	≥9.1% no HS diploma (Reference)	1.00	—	—
	<9.1% no HS diploma	0.98	0.97–0.99	0.001
**Region Type**	Metropolitan (Reference)	1.00	—	—
	Non-metropolitan	0.98	0.96–0.99	0.017
**Charlson–Deyo Score**	0 (Reference)	1.00	—	—
	1	0.84	0.83–0.85	<0.001
	2	0.78	0.76–0.80	<0.001
	3	0.71	0.69–0.73	<0.001
**Insurance**	Private (Reference)	1.00	—	—
	Government	0.87	0.86–0.88	<0.001
	Uninsured	0.90	0.87–0.93	<0.001
**Region**	Northeast (Reference)	1.00	—	—
	South	0.97	0.96–0.98	<0.001
	Midwest	1.04	1.02–1.06	<0.001
	West	0.97	0.95–0.98	0.012
**Histology**	Glioblastoma, NOS (Reference)	1.00	—	—
	Giant cell glioblastoma	1.22	1.13–1.31	<0.001
	Gliosarcoma	0.99	0.94–1.04	0.584
	Glioblastoma, IDH-mutant	1.73	1.57–1.91	<0.001

**Laterality**	Unilateral (Reference)	1.00	—	—
	Bilateral/Midline	0.87	0.86–0.88	<0.001

Abbreviations: Sx: Surgery; RT: Radiation Therapy; Chemo: Chemotherapy; TR: Time Ratio; CI: Confidence Interval; NOS: Not Otherwise Specified; IDH: Isocitrate Dehydrogenase; GBM: Glioblastoma Multiforme; Metro: Metropolitan area; Non-metro: Non-metropolitan area; HS: High School; Charlson–Deyo Score: Comorbidity index based on ICD codes; n (%): Frequency and percentage.

Table S12. Multivariable Log-Logistic Accelerated Failure Time Model for Overall Survival.

This model estimates the time ratio (TR) for overall survival among patients with glioblastoma, adjusting for demographic, clinical, socioeconomic, and treatment-related covariates. A TR greater than 1 indicates prolonged survival relative to the reference category, while a TR less than 1 indicates shorter survival.

Variable	Category	Time Ratio (TR)	95% CI	P-value
**Treatment**	Sx+RT+Chemo (Reference)	1.00	—	—
	RT+Chemo	0.55	0.54–0.56	<0.001
	RT+Surgery	0.56	0.55–0.57	<0.001
	Surgery alone	0.26	0.25–0.26	<0.001
	Other therapies	0.39	0.38–0.40	<0.001
	No treatment	0.15	0.14–0.15	<0.001
**Age**	<65 years (Reference)	1.00	—	—
	≥65 years	0.70	0.69–0.71	<0.001
**Sex**	Male (Reference)	1.00	—	—
	Female	1.05	1.04–1.06	<0.001
**Race**	White (Reference)	1.00	—	—
	Non-White	1.19	1.17–1.21	<0.001
**Ethnicity**	Non-Hispanic (Reference)	1.00	—	—
	Hispanic	1.23	1.19–1.26	<0.001
**Facility Type**	Academic (Reference)	1.00	—	—
	Community	0.90	0.89–0.91	<0.001
	Network	0.90	0.89–0.91	<0.001
**Income Group**	<$74,063 (Reference)	1.00	—	—
	≥$74,063	1.12	1.11–1.13	<0.001
**Education**	≥9.1% no HS diploma (Reference)	1.00	—	—
	<9.1% no HS diploma	0.99	0.98–1.00	0.174
**Region Type**	Metropolitan (Reference)	1.00	—	—
	Non-metropolitan	0.99	0.97–1.01	0.192
**Charlson–Deyo Score**	0 (Reference)	1.00	—	—
	1	0.85	0.84–0.86	<0.001
	2	0.78	0.76–0.80	<0.001
	3	0.72	0.70–0.74	<0.001

**Insurance**	Private (Reference)	1.00	—	—
	Government	0.87	0.86–0.88	<0.001
	Uninsured	0.89	0.86–0.92	<0.001
**Region**	Northeast (Reference)	1.00	—	—
	South	0.98	0.97–0.99	0.005
	Midwest	1.03	1.01–1.05	0.001
	West	0.99	0.97–1.01	0.249
**Histology**	Glioblastoma, NOS (Reference)	1.00	—	—
	Giant cell glioblastoma	1.18	1.10–1.27	<0.001
	Gliosarcoma	0.99	0.94–1.03	0.475
	Glioblastoma, IDH-mutant	1.72	1.58–1.88	<0.001
**Laterality**	Unilateral (Reference)	1.00	—	—
	Bilateral/Midline	0.87	0.86–0.88	<0.001

Abbreviations: Sx: Surgery; RT: Radiation Therapy; Chemo: Chemotherapy; TR: Time Ratio; CI: Confidence Interval; NOS: Not Otherwise Specified; IDH: Isocitrate Dehydrogenase; GBM: Glioblastoma Multiforme; Metro: Metropolitan area; Non-metro: Non-metropolitan area; HS: High School; Charlson–Deyo Score: Comorbidity index based on ICD codes.

Supplementary Figure S1. Scaled Schoenfeld Residual Plots for Race, Insurance, and Laterality.

Residuals for race and laterality demonstrated flat lines near zero, supporting the proportional hazards assumption. A minor upward trend was observed for insurance status, suggesting that the hazard associated with insurance may slightly change over time, though the effect size appeared minimal given the large cohort.

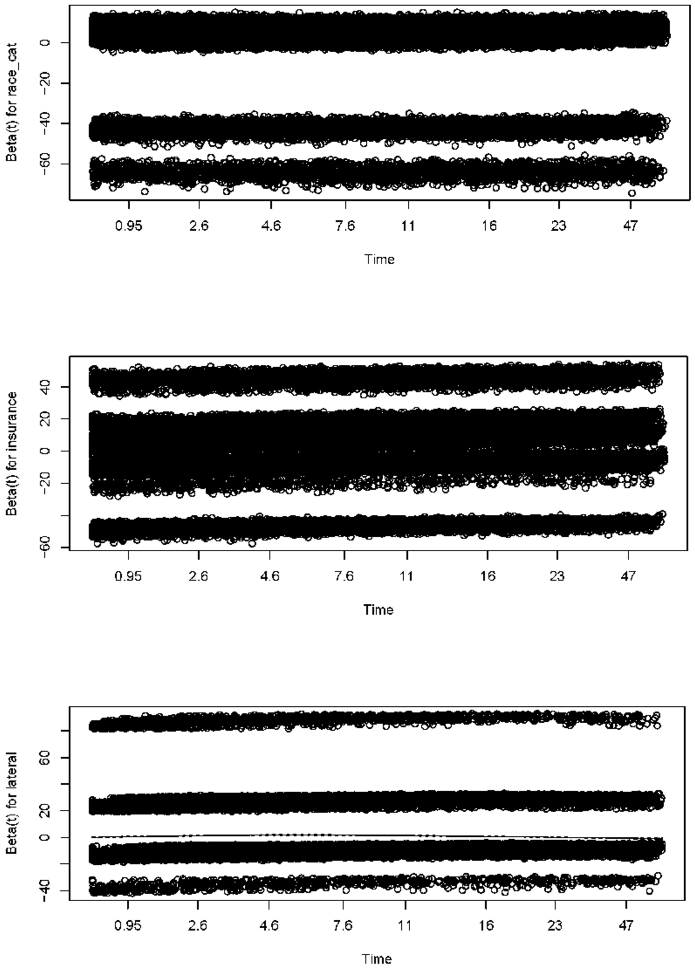

Supplementary Figure S2: Scaled Schoenfeld Residual Plots for Urbanicity (Metro/Non-Metro) and Location (Region).

Residuals for urbanicity (metro vs. non-metro) displayed a slight upward slope, indicating a modest time-varying effect, while geographic region (Northeast, South, Midwest, West) exhibited relatively stable curves, supporting proportionality.

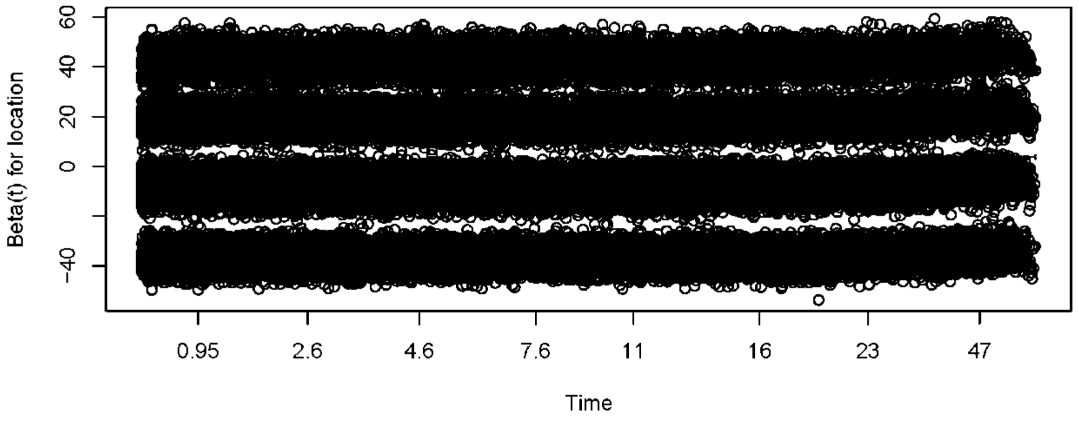

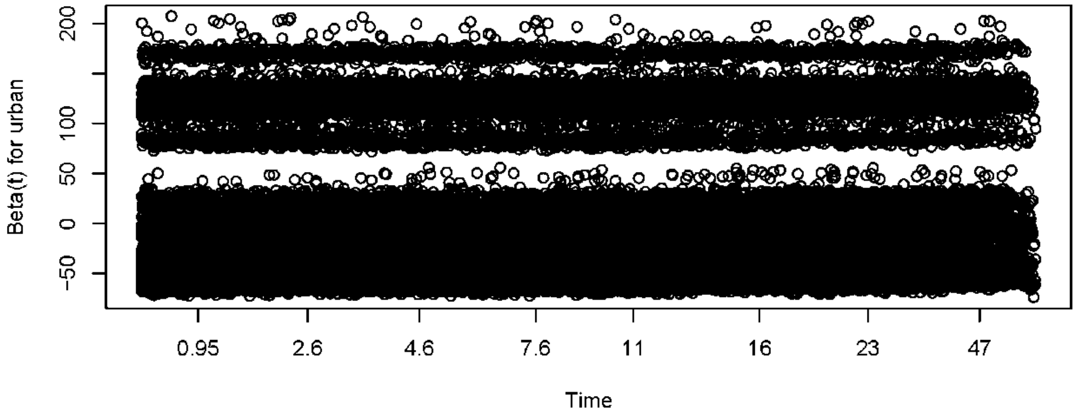

Supplementary Figure S3. Scaled Schoenfeld Residual Plots for Age, Treatment, and Diagnosis Year.

Residuals for treatment type showed a small early deviation with stabilization over time, suggesting that the effect of treatment was most pronounced early but remained generally proportional. Age and diagnosis year both demonstrated slight non-linearity but without strong departures from proportional hazards, consistent with expected shifts due to aging and evolving treatment paradigms.

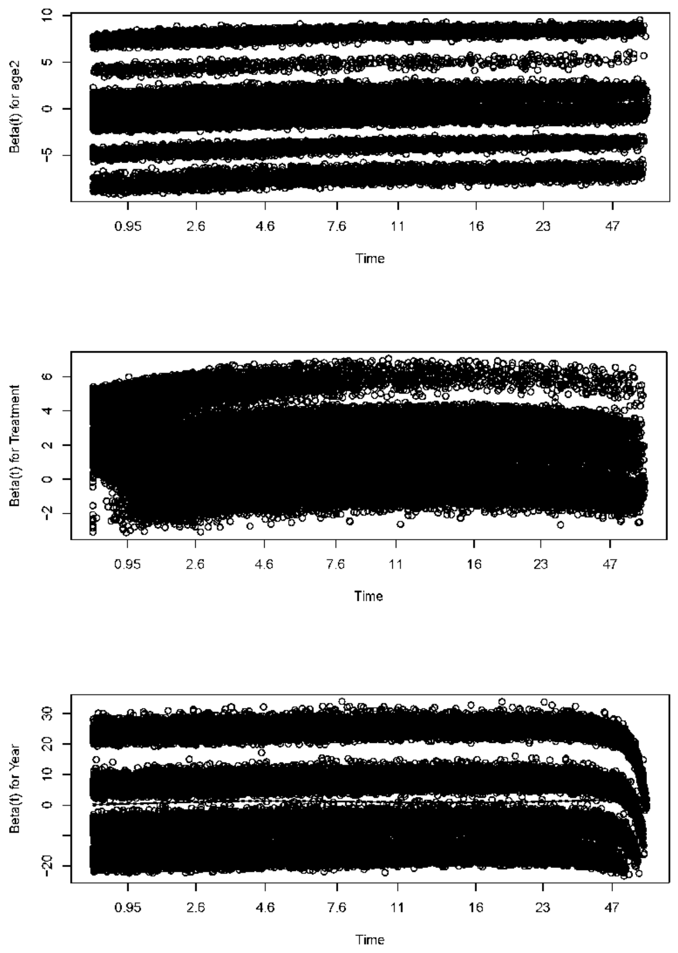

Supplement: Supplementary file 1 [file brainsci-15-00556-s001.zip › brainsci-3591290-supplementary.pdf]
